# An unusual cause of reversible complete heart block: right sinus of Valsalva rupture and compressive pseudo-aneurysm

**DOI:** 10.1093/ehjimp/qyad038

**Published:** 2023-11-08

**Authors:** Amélie Marang, Marc Bonnet, Thomas Rees, Jonathan Bentz, Sébastien Gerelli

**Affiliations:** Department of Cardiology, Centre Hospitalier Annecy Genevois, 1 avenue de l'Hôpital, Epagny Metz Tessy 74370, France; Department of Cardiology, Centre Hospitalier Annecy Genevois, 1 avenue de l'Hôpital, Epagny Metz Tessy 74370, France; Department of Cardiology, Centre Hospitalier Annecy Genevois, 1 avenue de l'Hôpital, Epagny Metz Tessy 74370, France; Department of Cardiovascular Surgery, Centre Hospitalier Annecy Genevois, 1 avenue de l'Hôpital, Epagny Metz Tessy 74370, France; Department of Cardiovascular Surgery, Centre Hospitalier Annecy Genevois, 1 avenue de l'Hôpital, Epagny Metz Tessy 74370, France

A 39-year-old man with no medical history presented to the emergency department with dizziness that had started 2 days earlier. Electrocardiogram showed complete heart block (*Figure 1A*).

**Figure 1:**
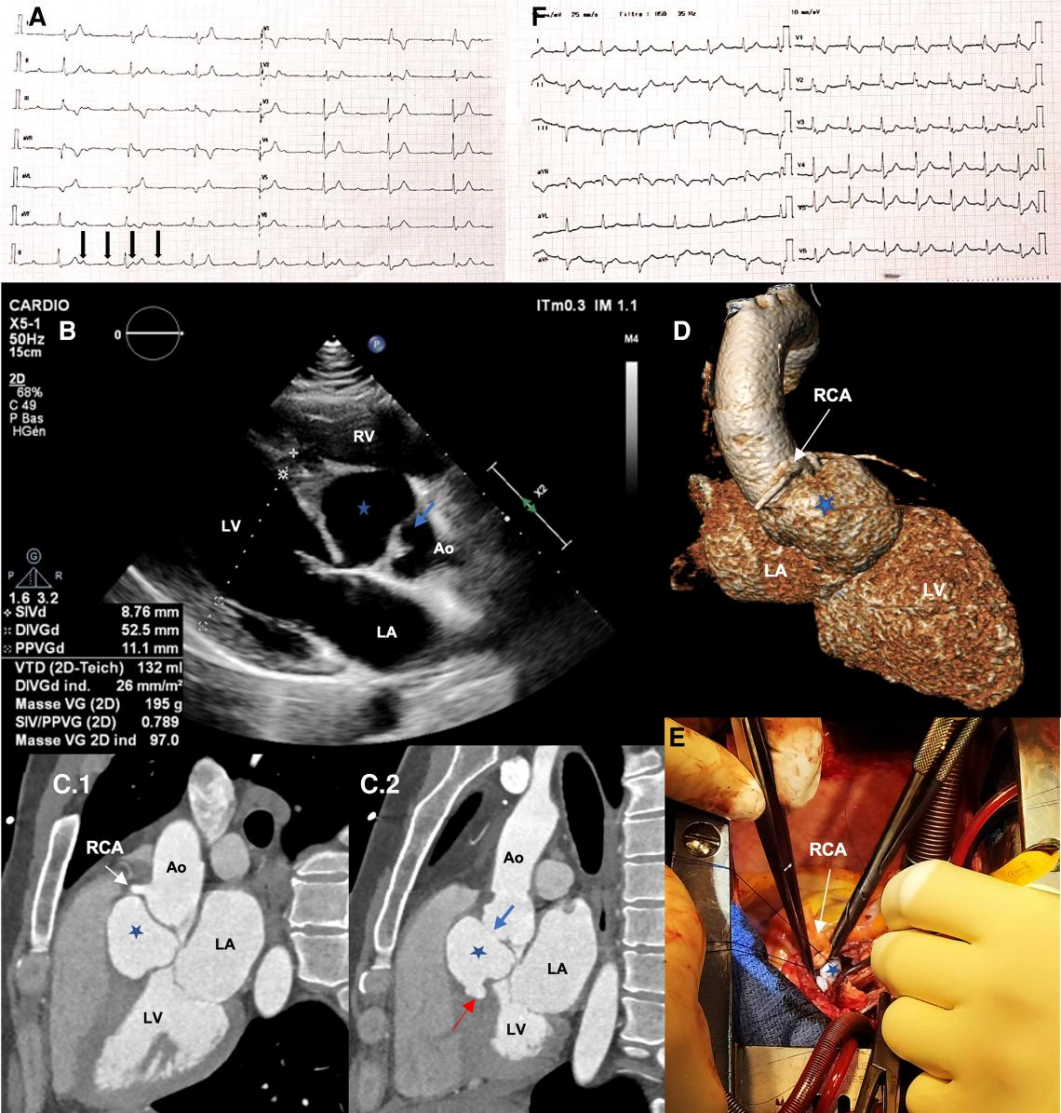
Electrocardiograms on admission and after surgery (panels *A* and *F* respectively), the parasternal long axis view of the transthoracic echocardiogram (panel *B*), cardiac CT and 3D reconstruction (panels *C* and *D* respectively) and pictures of the surgery after median sternotomy from the anesthesiologist's visual perspective (panel *E*). Blue star: pseudo-aneurysm; blue arrow: right sinus of Valsalva; red arrow: pseudo-aneurysm bleeding into interventricular septum. Ao, aorta; LA, left atrium; LV, left ventricle; RCA, right coronary artery.

The patient was haemodynamically stable on admission without isoprenaline, and physical examination was normal with no chest pain. Potassium and troponin levels were normal.

Transthoracic echocardiogram showed a pseudo-aneurysm (blue star) of the right sinus of Valsalva (blue arrow) without aortic dilatation or aortic regurgitation (*Figure 1B)*.

We performed a cardiac computed tomography (*Figure 1C* and *D*), which confirmed the rupture of the right sinus with a pseudo-aneurysm bleeding into the interventricular septum (red arrow).

The patient underwent emergency surgery with removal of the pseudo-aneurysm just below the right coronary artery (*Figure 1E*) and aortic root replacement with mechanical Bentall procedure.

Post-operative care was uneventful. We observed regression of the atrioventricular (AV) block on Day 2 with persistent right bundle branch block and left anterior fascicular block (*Figure 1F*).

The patient was discharged on Day 10 without the need for a pacemaker.

This case highlights an unusual reversible cause of AV block, which was most likely due to mechanical compression of the His bundle by a ruptured right coronary sinus aneurysm into the interventricular septum.

**Funding**: None declared.

## Data Availability

No new data were generated or analysed in support of this research.

## Lead author biography

My name is Amélie Marang. I am a cardiologist at the Centre Hospitalier d’Annecy Genevois in France. I graduated from medical school in 2017 at the Université Paris Descartes in Paris and then studied my specialty in cardiovascular medicine at Université de Tours, where I graduated in 2022. I am currently working as a non-invasive cardiologist in the cardiac intensive care unit and wish to specialize in cardiac critical care.

